# Determinants of life expectancy and clustering of provinces to improve life expectancy: an ecological study in Indonesia

**DOI:** 10.1186/s12889-020-8408-3

**Published:** 2020-03-18

**Authors:** Sekar Ayu Paramita, Chiho Yamazaki, Hiroshi Koyama

**Affiliations:** 1 Department of Public Health, Graduate School of Medicine, Gunma University, 3-39-22 Showa, Maebashi, Gunma 371-8511 Japan; 2 Department of Public Health, Universitas Padjadjaran, Jl. Eycman No.38, Bandung, 40161 Indonesia

**Keywords:** Life expectancy, Cluster analysis, Indonesia

## Abstract

**Background:**

Life expectancy acts as a population measure of the performance of healthcare systems. Regional disparities on life expectancy in Indonesia has been persisted and become a public health policy challenge. A systematic clustering of provinces can be a valuable alternative for organizing cooperation that aimed to increase life expectancy and reduce disparities. This study aimed to identify determinants of life expectancy and designate clusters of Indonesian provinces with similar characteristics. This approach can be useful in generating alternative cooperation strategies to improve life expectancy.

**Methods:**

We carefully selected variables that have been shown to impact life expectancy and gathered 2015 data from Indonesia’s Ministry of Health. All 34 Indonesian provinces were included as analysis units. We performed structural equation modeling (SEM) to select domains that needed to work on from theoretical models. Based on SEM results, we performed cluster analysis to arrange cooperation groups.

**Results:**

Life expectancy showed correlations with mean years of schooling, expenditure per capita, health workforce, healthcare facilities, and environment. Expenditure per capita also was the strongest of all constructs. Based on SEM results, we performed cluster analysis to arrange cooperation groups of total 34 provinces and generated five clusters of provinces.

**Conclusions:**

Enhancing the economy is the most effective approach for improving life expectancy and other constructs. These clusters can build cooperation that is new, within, and across clusters. These results may be useful in formulating cooperation strategies aimed at increasing life expectancy.

## Background

Life expectancy has been used to compare social categories within countries or to compare healthcare systems as a whole. It acts as a population measure of the performance of healthcare systems, and wellbeing of population [1]. Life expectancy of Indonesians between 1990 and 2016 has increased by approximately 8 years. However, provincial disparities on life expectancy persisted, for example, eastern provinces life expectancies are considerably lower compared to provinces in Java, Sumatra and Bali [2, 3]. Life expectancy disparities become a public health policy challenge.

In public health, it is known that addressing health goals through cooperation with others will make important strides [4]. Cooperation allows communities to help each other, act together, and align plans and priorities. Cooperation that aimed to achieve common goals has been approached in many different forms. For example, there are across country cooperation such as South-South cooperation [5, 6], North-South Sudan cooperation [7], European Union [8], the United Nations cooperation [9], etc. In Japan, prefectures with similar historic, cultural, and geographic backgrounds are grouped into regions, and these regions work together in many sectors, including health sector [10, 11]. Many provinces in Indonesia shared similar challenges and concerns to achieve public health solutions. A systematic clustering of provinces can be a valuable alternative for organizing cooperation. Provinces can be considered as working units that can work cooperatively in groups on a structured activity. Cooperative group can share knowledge and experiences to improve health. Cooperation can become an opportunity to learn and gain an understanding new perspective, which can be extremely valuable on achieving public health goals. However, there is no systematic clustering of Indonesian provinces yet.

Therefore, this study aimed to identify determinants of life expectancy and designate clusters of Indonesian provinces with similar characteristics. These clusters of provinces can form cooperative groups. These cooperative groups should work together on a public health policy related to life expectancy disparities.

## Methods

### Study preparation and data settings

The study is a secondary data analysis. We used 2015 published data from the Ministry of Health of Indonesia. This dataset is fully accessible for public without restrictions. All 34 Indonesian provinces were included as analysis units. Evaluation criteria were necessary to ensure the quality of the data. These criteria included the variables’ standard definition, methods of data collection, comparing data from different sources, and evaluating the 16-years trend of the respective variables to assess data accuracy.

### Structural equation modeling

Structural equation modeling (SEM) is a statistical modeling technique to describe relationships between theoretical constructs, represented by regression or path coefficients between the factors [12]. SEM could become an indispensable tool for managers, policymakers, and regulators in the healthcare sector [13]. It implies a structure for the covariance between the observed variables, and latent factors. SEM enables complex pathways to be tested simultaneously and focusing on relationships among underlying factors. It is a suitable statistical method to investigate variables associations under a theoretical model and allows us to test the validity of the model based on a set of measured variables in an attempt to explain their observed variances and covariance.

To create our theoretical model, we reviewed previous studies [14–17], government reports [18–22], and other literatures [16, 23–29]. We selected the variables that have most commonly been shown to impact life expectancy, especially in Indonesia.

Health system is the main support for health status [2, 14–16, 23–31]. Indonesian provinces that are underdeveloped faced more difficulties to access healthcare [18–22]. Differences of development on facilities may have caused of differences in health services [30] that will affect health. Provinces with lower number of healthcare facilities and health workforce per population have lower life expectancy rates [2]. Health workforce availability will assure of access to health services needed, securing the public health efforts that eventually will improve the health status of the community [30]. Insurance ownership creates an opportunity to get access to services, and further ease the financing to get access to and intensive medical care [30].

Socioeconomic and demographic factors also influence life expectancy [2, 14–17, 23–31]. Declining of income inequality contributes to increasing life expectancy in Indonesia [30]. Higher income per capita is associated with higher life expectancy [31]. Poverty has a strong relationship with the life expectancy in Indonesia [2]. In 2016, Indonesia’s poverty has declined from the previous year; this has contributed to increasing the life expectancy of the community in Indonesia. Education is also a determinant of life expectancy [17], the increase of mean years of schooling and expected years of schooling increase proportionally with life expectancy [18–22]. Environmental factors play a role in ensuring health. The increase of the percentages of households with clean water and percentages of households with proper sanitations increase proportionally with life expectancy in Indonesia [18–22].

In this study, we used SEM to test for pathways towards life expectancies to other structure in our theoretical model. A latent variable is a non-observed random variable that comprises two or more correlated measured variables. It cannot be measured directly and was estimated on the basis of observed variables. We hypothesized the latent variables have bilateral correlation toward each other and causal correlations with observed variables. We also hypothesized possible relationships of variables in theoretical models and considered all possible groupings of health determinants. Following on the literature review, we constructed four latent variables linked to life expectancy as follows:

1. Health system: (1) insurance ownership, (2) number of general physicians per population, (3) number of specialist physicians per population, (4) number of nurse per population, (5) number of midwifes per population, (6) number of *puskesmas* (community health center) per population, (7) number of hospital per population, and (8) number of hospital beds per population.
2. Socioeconomic: (1) expected years of schooling, (2) mean years of schooling, (3) Gini index (income), (4) poverty, and (5) expenditure per capita.
3. Demographics: (1) maternal mortality ratio, and (2) infant mortality ratio.
4. Environment: (1) percentage of households with clean water and (2) percentage of households with proper sanitation.

The theoretical model was tested in Lavaan package in RStudio version 1.0.136 –© 2009–2016 RStudio, Inc. using the maximum likelihood estimator. We assess and modify theoretical models to find the best-fit model. The interpretability of the factors was also evaluated and considered during comparison of the models. Goodness of fit of the final model was evaluated with fit indices such as chi-square, comparative fit index (CFI), and standardized root mean square residual (SRMR). These indices provide different information about model fit allowing for a more conservative and reliable evaluation of the model. To achieve a good-fit model, we simplified latent variables, segregated “health system” into three latent variables: “health insurance”, “health workforce” and “healthcare facilities”, and statistically insignificant paths were removed based on their *P*-values < 0.05. Establishing a good-fit model is important to select legitimate variables to be included in cluster analysis.

### Cluster analysis

We used k-means cluster analysis to reveal natural clusters [32–34] of provinces based on variables in the final model. We used elbow method to determine the most appropriate number of clusters [35]: we computed values of k varying from two to seven clusters, calculate the total within-cluster sum of squares (wss), and plot the curve of wss based on the number of clusters k. The bend in the plot was located in the five-cluster wss; therefore, we chose to use five clusters.

## Results

### SEM

**Fig. 1:**
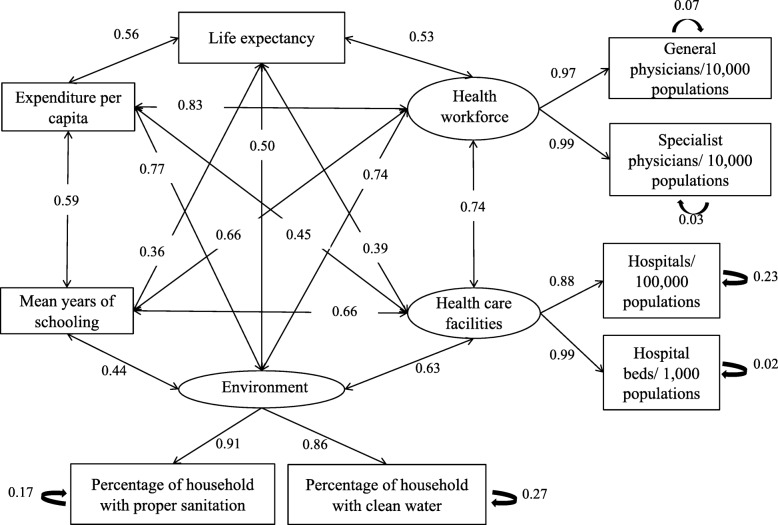
Final model describing the relationship between life expectancy, health workforce, healthcare facilities, the environment, mean years of schooling, and expenditure per capita. The fit between the model and the data was adequate: chi-square 0.005; CFI 0.935; and SRMR 0.054. Source: Author’s (SAP) analysis of the Health Profile of Indonesia for 2015

We achieved adequate fit after 133 iterations (chi-square 0.005; CFI 0.935; and SRMR 0.054). In the final model (Fig. 1), there were nine observed variables life expectancy, general physicians/10,000 people, specialist physicians/10,000 people, hospital/100,000 people, hospital beds/1000 people, percentages of households with clean water and percentage of households with proper sanitation, mean years of schooling and expenditure per capita; and three latent variables: health workforce, healthcare facilities, and environment. “Health workforce” was a latent variable for general physicians/10,000 people (0.97) and specialist physicians/10,000 people (0.99). “Healthcare facilities” was a latent variable for hospital/100,000 people (0.88) and hospital beds/1000 people (0.99). “Environment” was a latent variable for percentages of households with clean water (0.86) and percentage of households with proper sanitation (0.91).

From all variables, there were six constructs with bilateral correlations towards each other: (1) life expectancy, (2) health workforce, (3) healthcare facilities, (4) environment, (5) mean years of schooling, and (6) expenditure per capita. Magnitude of correlation between six constructs ranged from 0.83 (health workforce and expenditure per capita) to 0.36 (life expectancy and education). Life expectancy bilateral correlations from strongest to weakest correlation was with expenditure per capita (0.56), health workforce (0.53), environment (0.5), healthcare facilities (0.39), and mean years of schooling (0.36). Expenditure per capita bilateral correlations from the strongest to weakest correlation, respectively, was with environment (0.77), mean years of schooling (0.59), life expectancy (0.56), and healthcare facilities (0.45). The rest of bilateral correlation among constructs were built among health workforce and healthcare facilities (0.74), health workforce and environment (0.74), health workforce and mean years of schooling (0.66), healthcare facilities and mean years of schooling (0.66), healthcare facilities and environment (0.63), and environment and education (0.44).

### Cluster analysis

**Fig. 2:**
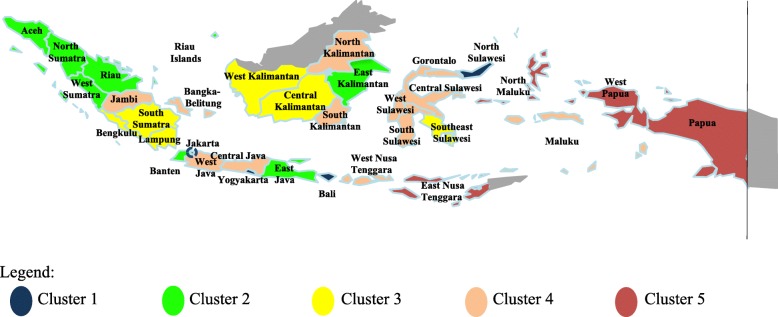
Map of Indonesia based on cluster analysis results. Source: Author’s (SAP) analysis of the Health Profile of Indonesia for 2015. Figure was drew and colored by Author (SAP) using Microsoft Power Point for Mac

**Fig. 3:**
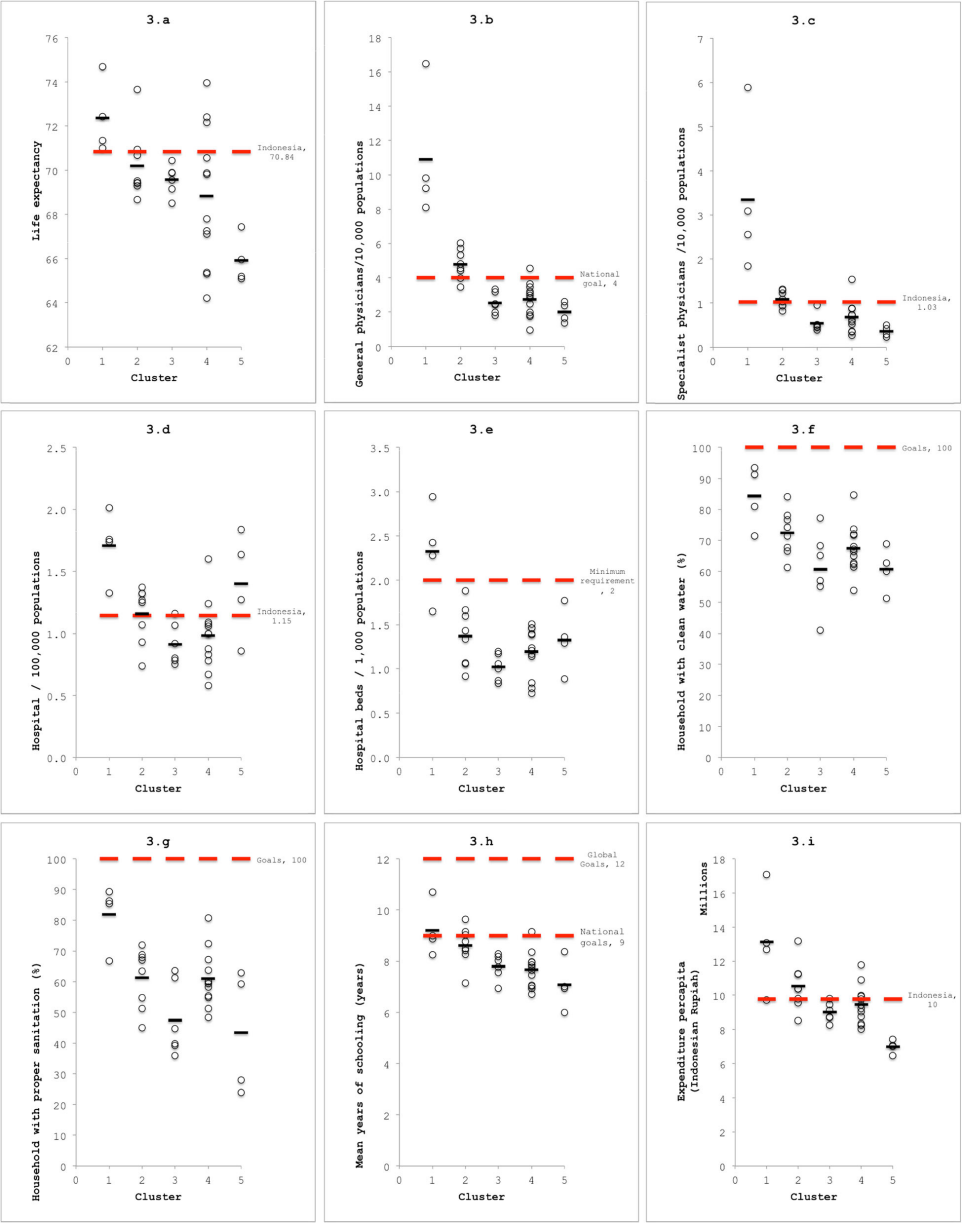
Provincial data based on cluster analysis results compared with national footing. Source: Author’s (SAP) analysis of the Health Profile of Indonesia for 2015

Five clusters of provinces were generated (see Table 1, Fig. 2, and Fig. 3). We sorted these based on the respective best to worst inclusive characteristics.

**Table 1:** Cluster analysis results

Cluster 1	Cluster 2	Cluster 3	Cluster 4	Cluster 5
Best inclusive characteristics	Good inclusive characteristics	Average inclusive characteristics	Poor inclusive characteristics	Worst inclusive characteristics
**Province**				
1. Jakarta 2. Yogyakarta 3. Bali 4. North Sulawesi	1. Aceh 2. North Sumatera 3. West Sumatera 4. Riau 5. Riau Islands 6. East Java 7. Banten 8. East Kalimantan	1. South Sumatera 2. Bengkulu 3. Lampung 4. West Kalimantan 5. Central Kalimantan 6. Southeast Sulawesi	1. Jambi 2. Bangka Belitung 3. West Java 4. Central Java 5. West Nusa Tenggara 6. South Kalimantan 7. North Kalimantan 8. Central Sulawesi 9. South Sulawesi 10. Gorontalo 11. West Sulawesi 12. Maluku	1. East Nusa Tenggara 2. North Maluku 3. West Papua 4. Papua

Source: Author’s (SAP) analysis of the Health Profile of Indonesia for 2015

Figure 3a shows the average life expectancy in 2015 for Indonesian citizens was 70.84 years. All provinces in cluster 1 had life expectancies above the national average (range 70.99–74.68 years, mean 72.36 years), while cluster 2 had an average value (range 68.66–73.65 years, mean 70.19). All provinces in cluster were below the average (range 68.5–70.44 years, mean 69.57). Cluster 4 had the widest range (range 64.22–73.96 years, mean 68.82). Cluster 5 had the collectively lowest (range 65.09–67.44 years, mean 65.92). Life expectancy in all cluster 5 provinces was below the Indonesia’s average.

Figure 3b shows Indonesia’s national goal for number of general physicians/10,000 people was four general physicians/10,000 people. All provinces in cluster 1 exceeded the goal for number of general physicians per populations (range 8.10–16.44, mean 10.89). Most in cluster 2 exceeded it (range 3.46–6.03, mean 4.79), while a majority in cluster 4 still below the goal (range 0.96–4.55, mean 2.73). The number of general physicians/10,000 people in all provinces in cluster 3 (range 1.80–3.33, mean 0.54) and cluster 5 (range 1.37–2.60, mean 2.00) remained below the goal.

Figure 3c shows Indonesia’s average number of specialist physicians/10,000 people was 1.03. All provinces in cluster 1 exceeded the national average (range 1.84–5.89, mean 3.34), while all provinces in cluster 3 (range 0.39–0.96, mean 0.54) and cluster 5 (range 0.22–0.50, mean 0.36) ranked among the lowest, both cluster were below the national average. Cluster 2 showed average numbers (range 0.82–1.31, mean 1.08). In cluster 4 (range 0.27–1.54, mean 0.68), only one province (South Sulawesi) surpassed the national average, while other 11 provinces were below it.

Figure 3d shows that Indonesia’s average for number of hospitals per 10,000 populations was 1.15. Highest to lowest averages were cluster 1 (mean 1.71), cluster 5 (mean 1.40), cluster 2 (mean 1.16), cluster 4 (mean 0.98), and cluster 3 (mean 0.91). All provinces in cluster 1 exceeded the national average (range 1.32–2.01). Cluster 4 had the widest variations of number of hospitals/10,000 people (range 0.58–1.60). Figure 3e shows 31 of 34 provinces were below the minimum requirement from Word Health Organization (WHO) of two hospital beds/10,000 people. Cluster 1 (mean 2.33) had the highest average, followed in order by clusters 5 (mean 1.33), cluster 2 (mean 1.37), cluster 4 (mean 1.19), and cluster 3 (mean 1.02).

Figure 3f and g show the percentages of households with clean water and percentages of households with proper sanitations in all provinces in all clusters were below 100%. Cluster 1 (mean 84.31) had the highest average for the percentages of households with clean water, followed in order by cluster 2 (mean 72.50), cluster 4 (mean 67.42), cluster 5 (mean 60.73), and cluster 3 (mean 60.65). A province in cluster 3 had the lowest percentage of households with clean water (41%). For percentages of households with proper sanitations in all provinces, the highest average also belongs to cluster 1 (mean 81.96), followed in order by cluster 2 (mean 61.28), cluster 4 (mean 60.98), cluster 3 (mean 47.44), and cluster 5 (mean 43.48). Two provinces in cluster 5 were lowest (28 and 23%).

Figure 3h shows there are two goals for mean years of schooling: global (12 years), and national (9 years). None of the 34 provinces achieved the global goals. Cluster 1 (range 8.26–10.70, mean 9.21) had the highest mean years of schooling, followed by cluster 2 (range 7.14–9.65, mean 8.62), cluster 3 (range 6.93–8.29, mean 7.79), cluster 4 (range 6.71–9.16, mean 7.66), and cluster 5 (range 5.99–8.37, mean 7.08). None of the provinces in cluster 3 and cluster 5 achieved national goals.

Figure 3i shows Indonesia’s average expenditure per capita was 10 billion Indonesian rupiahs per year. Cluster 1 was highest (mean 13.1 billion rupiahs), followed in order by cluster 2 (mean 10 billion rupiahs), cluster 4 (mean 9.4 billion rupiahs), cluster 3 (mean 9 billion rupiahs), and cluster 5 (mean 6 billion rupiahs). All provinces in cluster 5 had the lowest expenditure per capita (range 6.4–7.4 billion rupiahs) compared with provinces in other clusters.

## Discussion

The results from the SEM in the present study are potentially useful for understanding the relationship among variables, set priorities, and can be useful toward designing organized cooperation strategies. From this SEM, we found expenditure per capita was strongest among the six constructs. This implies that enhancing the economy is the most effective approach to improving life expectancy and other constructs. Indonesia’s gross domestic product per capita has steadily risen, from $857 in 2000 to $3603 in 2016 [36]. However, among the country’s 252 million people, more than 28 million still live below the national poverty line [37]. Further, approximately 40% of the entire population remains vulnerable to falling into poverty, as their incomes are only marginally above the national poverty line [36]. The annual reports of the Ministry of Health have consistently shown unequal distribution of healthcare resources in the country [19–22, 38–43], and our previous study validated this [44]. Inequality in Indonesia undermines the fight against poverty, serving as a brake on economic growth and threatening social cohesion [45]. To strengthen the investment climate and bolster economic growth, fiscal policies should be aimed at strengthening tax collection and broadening the tax base through tax reform [36].

The results of cluster analysis in this study may be useful as a guide to improving coordination between provincial and national governments, while leveraging regional integration. Progress of current efforts on reducing life expectancy disparities in Indonesia seemed slow-moving. We viewed that application of the results on this study can add new perspectives. These exchanges have the potential to impact provincial integration processes and health policy debates. Cooperation among provinces may strengthen, share and accelerate health development within and across clusters. Results of this study is useful to assemble cooperation within clusters and across clusters. Provinces with similar characteristics will have similar goals and priorities. These provinces can work together in terms that are best suited to their characteristics. The clusters of provinces can build new cooperative groups and proceed to work together. The national government should support local governments, especially in provinces within the more economically challenged clusters.

This study has one notable limitation. The study subjects were the provinces; therefore, the number of observations were inevitably only 34. Nonetheless, this limited number of observations did not prevent us from achieving adequate fit for the final model.

## Conclusions

The results of this study provide evidence that expenditure per capita is the core factor in improving life expectancy, as are health workforce, healthcare facilities, the environment, and mean years of schooling. Expenditure per capita is also an important component for clustering of the provinces. This clustering of provinces will make it easier to organize cooperation within and across clusters. Provinces in the same cluster have similar characteristics and therefore should have similar goals and priorities. These provinces can work together in terms that are best suited to their characteristics. The clusters can build new cooperative groups and thereby work together. The national government should support local governments, especially in provinces within the more economically challenged clusters.

## Data Availability

We used 2015 published data from the Ministry of Health of Indonesia. We confirm that all data is fully accessible for public without restrictions.

## Abbreviations

CFI: Comparative fit index
SEM: Structural equation modeling
SRMR: Standardized root mean square residual
WHO: Word Health Organization
wss: Within-cluster sum of squares
